# Passive Deicing CFRP Surfaces Enabled by Super-Hydrophobic Multi-Scale Micro-Nano Structures Fabricated via Femtosecond Laser Direct Writing

**DOI:** 10.3390/nano12162782

**Published:** 2022-08-13

**Authors:** Zihan Zhang, Jiakang Zhou, Yuqi Ren, Weihan Li, Sheng Li, Nianyao Chai, Zhongle Zeng, Xiangyu Chen, Yunfan Yue, Ling Zhou, Yibing Cheng, Shuxin Li, Xuewen Wang

**Affiliations:** 1 State Key Laboratory of Advanced Technology for Materials Synthesis and Processing, International School of Materials Science and Engineering, Wuhan University of Technology, Wuhan 430070, China; 2 Foshan Xianhu Laboratory of the Advanced Energy Science and Technology Guangdong Laboratory, Foshan 528216, China; 3 Hubei Key Laboratory of Theory and Application of Advanced Materials Mechanics, School of Science, Wuhan University of Technology, Wuhan 430070, China

**Keywords:** femtosecond laser structuring, CFRP, multi-scale micro-nano structure surface, super-hydrophobic, anti-icing application

## Abstract

Carbon fiber reinforced plastic (CFRP) is the main material of aircraft skin. Preparing superhydrophobic anti-icing/deicing surface on the CFRP is of great importance for aircraft flight safety. In this work, a variety of multi-scale micro-nano structures were imprinted on CFRP by femtosecond laser processing, and a transition from hydrophilic to superhydrophobic CFRP was realized. After being optimized by different geometries and laser conditions, the water contact angle, which is tested at 24.3 °C and 34% humidity, increased from 88 ± 2° (pristine) to 149 ± 3° (100 μm groove) and 153 ± 3° (80 μm grid). A further anti-icing test at −10 °C (measured on the cooling platform) and 28% humidity showed that the freezing time was increased from 78 ± 10 s (pristine) to 282 ± 25 s (80 μm grid). Most importantly, the tensile tests showed that the femtosecond laser processing method did not deteriorate the mechanical properties of CFRP. This work provides great significance for aircraft passive deicing technology.

## 1. Introduction

Icing will affect the aerodynamic performance of aircraft, reduce flight stability and safety, and cause damage to the aero engine, resulting in serious accidents [1,2,3]. Aircraft anti-icing/deicing technology has become an important consideration in the aviation industry [4,5,6]. Traditional anti-icing methods are mainly active deicing with additional treatments including thermal, mechanical, and chemical methods. However, these methods often have drawbacks of low efficiency, high energy consumption, and even damage to the surface of the fuselage, leading to potential dangers during flight [7]. Therefore, it has great potential to fabricate superhydrophobic structures on the surface of the fuselage skin to achieve passive anti-icing. Inspired by the superhydrophobic structure of lotus leaves and roses, preparing a bionic multi-scale micro-nano structure on the surface of the fuselage may prevent water droplets from sticking to the surface of the aircraft during flight, delay the freezing time and achieve an anti-icing/deicing effect. With these micro-nano structures, the contact angle (CA) between the droplet and the material can reach more than 150° (superhydrophobic state). Additionally, the contact area between the droplet and the material surface is limited, which helps the droplets fall off from the surface and avoids ice formation during high-speed flight [8]. At the same time, the bubbles captured on the superhydrophobic surface also delay the non-uniform nucleation time of the droplets and prevent the freezing process [9]. In addition, superhydrophobic surfaces are also widely used in antibacterial, anticorrosion, and other fields [10].

Carbon fiber reinforced plastics (CFRP), because of their characteristics of light weight, high strength, stiffness, and elastic modulus are widely used as aircraft skin materials. Therefore, preparing superhydrophobic micro-nano structures on CFRP and then bonding the CFRP on the surface of the leading edge of the wing will achieve an anti-icing surface on complex structures. Anti-icing using carbon materials has been reported, Naureen Akhtar et al. studied the fluorination of graphene under high humidity (50–55%) to significantly enhance the anti-icing performance, which can be delayed for 90 min at −15 °C and even up to 6 h and 45 min at −5 °C [11]. Signe Kyrkiebo et al. studied the anti-icing phenomenon of graphene/graphene oxide grown on the Ir(111) surface and found that graphene oxide would have a lower freezing onset temperature than pristine graphene [12]. Both of these indicate that surface functionalization can significantly improve the anti-icing performance of carbon nanomaterials. So, how to achieve functionalization or structurization is the key to the preparation of anti-icing or superhydrophobic surfaces. The techniques for preparing superhydrophobic surfaces mainly include the template method [13], electrochemical deposition method [14], etching method [15], and sol–gel method [16]. However, these methods usually use complex chemical reagents or have complicated procedures with low efficiency and limited material size [17]. In recent years, the rapid development of ultrafast laser processing technology provides an efficient way to prepare micro-nano structures on various material surfaces. With this method, the scribing process is microstructured but the scribing process produces nanoparticles on the rim of the scribed line. Compared with traditional methods, ultra-fast laser processing has significant advantages in efficiency, precision, and flexible control method [18,19]. Kietzi used a femtosecond laser to etch micro-nano structures on the surfaces of different alloys [20]. Water droplets could be completely spread out on the surface of the newly treated alloys, presenting a superhydrophilic state. However, after the surface was placed in the air for a certain period of time, the surface eventually reached the superhydrophobic state due to the absorption of carbon elements in the air by the surface. Ge et al. used femtosecond laser technology to construct a Siberian-Cocklebur-like microstructures surface on polytetrafluoroethylene (PTFE) substrates for anti-icing application. The freezing time of the treated surface was about two times of the pristine counterpart [21]. Yang et al. used picosecond laser direct writing and the fluoroalkylsilane immersion method to functionalize CFRP, and the functionalized surface possesses a static water contact angle > 150° and rolling-off angle < 10° [22]. Considering the non-ignorable thermal effect during laser–matter interactions, there will be limited thermal damage to CFRP if the laser pulse is much shorter than the thermal diffusion time scale.

Therefore, we developed and optimized the femtosecond laser manufacturing process of multi-scale micro-nano structure on CFRP and obtained a super-hydrophobic surface with the aim to meet the requirements of anti-icing of key components such as the leading edge of the wing. A variety of trans-scale micro-nano structures, such as micro grooves and grids were prepared and an obvious transition from hydrophilic to superhydrophobic was realized. For example, the water contact angle was increased from 88 ± 2° (pristine) to 149 ± 3° (100 μm groove) and 153 ± 3° (80 μm grid). Furthermore, the freezing time was delayed from 78 ± 10 s (pristine) to 282 ± 25 s (80 μm grid) in the icing test (−10 °C and 28% humidity). This work provides an effective way of anti-icing and deicing applications.

## 2. Experimental Section

The CFRP with 0.4~0.5 mm in thickness was provided by Commercial Aircraft Corporation of China Ltd. (COMAC). Firstly, the CFRP was cut into small pieces with 30 × 30 mm^2^. After cleaned by ultrasonic in ethanol for 5 min and dried, the CFRP was carved by a 1030 nm laser with Pharos of Galvo scanning model in which two mirrors were used to control the laser to scan and process in the x and y directions. The pulse width was 500 fs. The radius of the focal spot produced by the Gaussian beam was about 25 μm.

The processed samples were blown with nitrogen gas to remove the chippings. Then, samples were immersed in 2% 1H,1H,2H,2H-Perfluorooctane trimethoxysilane (PFDTS)—isopropyl alcohol solution for 2 h and dried at 70 °C for 1 h. The surface morphology was checked by a TESCAN MIRA LMS scanning electron microscope (SEM). The water contact angle was measured by a contact angle meter (Biolin Theta Lite) where the temperature is 24.3 °C and humidity is 34%. The volume of each water droplet was 6 μL. Then, the samples were put on a cooling platform and the freezing behavior of water droplets (4 μL) on CFRP surfaces with different morphologies was recorded by a CCD camera. To measure the mechanical properties, the CFRP was cut into the standard dumbbell-shaped sample with the tested region of 20 (length) × 4 (width) mm^2^. Then, a universal testing machine (INSTRON 5848 Micro Tester) was used to test the tensile tests with a 5 mm/min loading rate.

## 3. Results and Discussions

**Figure 1:**
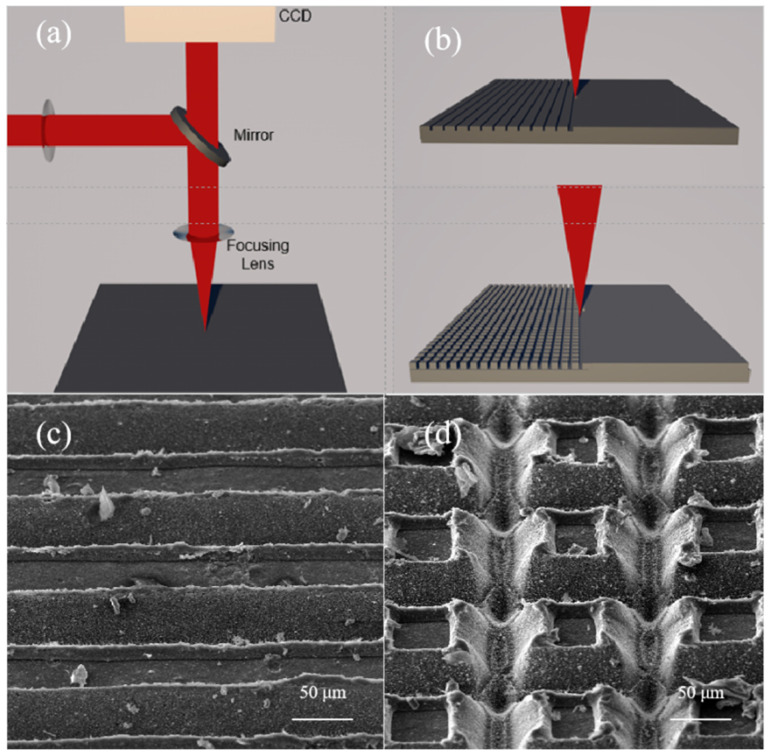
(**a**) Schematic of the laser machining system, (**b**) two types of scanning mode, (**c**) a 45° field of view SEM figure of grooves with 80 μm spacing, and (**d**) a 45° field of view SEM figure of grids with 100 × 100 μm^2^ spacing.

The schematic of the experimental device is shown in Figure 1a. The laser beam is emitted and then modulated before the spectroscope. Then, the laser beam is sent to the CCD and the sample using a beam splitter. One is received by the charge-coupled CCD camera for detection and the other is focused on the sample for processing. In this experiment, according to the hydrophobic structure of nature, we constructed a layer of micro-nano protrusions and nanoparticles on the surface of CFRP. Two scanning modes, grooves (upper: writing parallel lines) and grids (lower: writing parallel lines rotated 90 deg) were designed to process, as shown in Figure 1b. Figure 1c,d show the SEM figures (45° field of view) of surface morphology of grooves (80 μm space, repetition rate = 200 kHz, scanning speed = 100 mm/s, laser power density = 3.8 × 10^4^ W/cm^2^) and grids (100 × 100 μm^2^ spacing, repetition rate = 200 kHz, scanning speed = 100 mm/s, laser power density = 5.7 × 10^4^ W/cm^2^).

In order to optimize the process parameters, grooves with spacing from 60 μm to 160 μm and grids with 80 × 80 μm^2^ to 180 × 180 μm^2^ at a fixed increment of 20 μm were written on the CFRP. The laser power density is 3.8 × 10^4^ W/cm^2^ for grooves and 5.7 × 10^4^ W/cm^2^ for grids, respectively. The repetition rate is fixed at 200 kHz and the scanning speed is fixed at 100 mm/s according to the previous optimization work.

**Figure 2:**
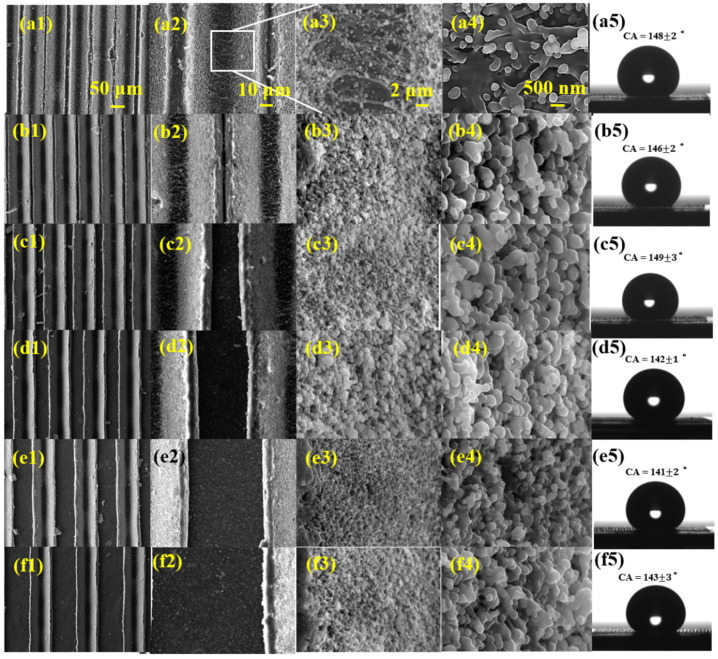
SEM images of groove structure with different spaces and the corresponding contact angle images. (**a1**–**a5**) Shows 60 μm spacing, CA = 148 ± 2°, (**b1**–**b5**) 80 μm spacing, CA = 146 ± 2°, (**c1**–**c5**) 100 μm spacing, CA = 149 ± 3°, (**d1**–**d5**) 120 μm spacing, CA = 142 ± 1°, (**e1**–**e5**) 140 μm spacing, CA = 141 ± 2°, (**f1**–**f5**) 160 μm spacing, CA = 143 ± 3°. The laser power density is 3.8 × 10^4^ W/cm^2^.

Figure 2 shows the SEM images of the groove structure with different spacing and the corresponding contact angle images. Figure 2a1–a4 indicates the surface morphologies with a different magnification of the groove structure with 60 μm spacing. The grooves with clear and uniform edges were obviously observed in Figure 2a1. The top width of the groove is 80 μm and the bottom width is about 25 μm. From the magnified figure in Figure 2a3,a4, large amounts of nanoparticles are observed on the scribed surface. Thus, multi-scale micro-nano structure surfaces, including micro grooves and nanoparticles, were successfully processed on CFRP by femtosecond laser processing.

From Figure 2a1–f1, groove spaces increased from 60 μm to 160 μm as designed, while the nanoparticles on the slopes remained in a nearly unchanged size. The measured contact angles of the corresponding surface morphology are shown in Figure 2a5–f5. The contact angle is 148 ± 2° for the groove structure with 60 μm spacing, then the value changes with the groove spacing, and changes to 143 ± 3° for the 160 μm spacing groove surface.

**Figure 3:**
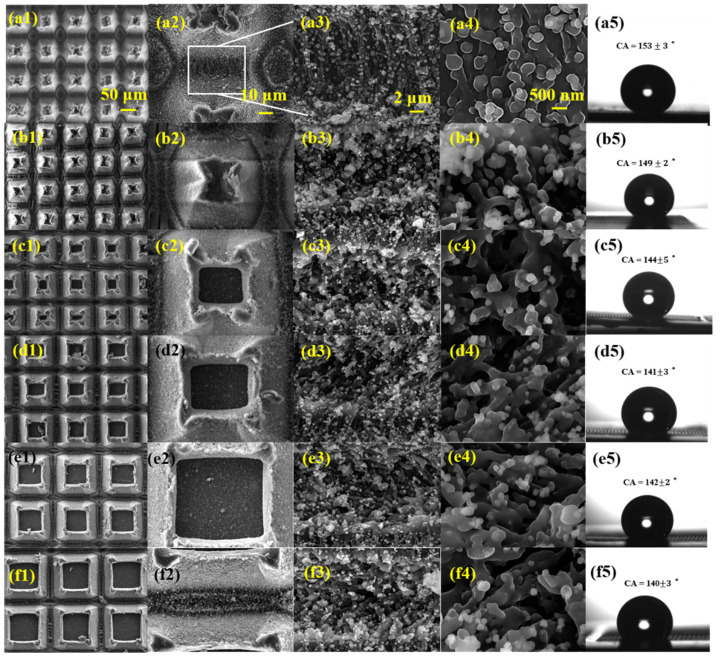
SEM images of grid structure with different spaces and the corresponding contact angle images. (**a1**–**a5**) Shows 80 μm space, CA = 153 ± 3°, (**b1**–**b5**) 100 μm space, CA = 149 ± 2°, (**c1**–**c5**) 120 μm space, CA = 144 ± 5°, (**d1**–**d5**) 140 μm space, CA = 141 ± 3°, (**e1**–**e5**) 160 μm space, CA = 142 ± 2°, (**f1**–**f5**) 180 μm space, CA = 140 ± 3°. The laser power density is 5.7 × 10^4^ W/cm^2^.

Figure 3 shows the SEM images of the grid structure with different spaces and the corresponding contact angle images. The laser power density is 5.7 × 10^4^ W/cm^2^. Figure 3a1–f1 shows the grids from 80 × 80 μm^2^ to 180 × 180 μm^2^ in size. These grids are actually micro columns that are narrow at the top and wide at the bottom, and their sides are covered with many nanoparticles, just like the grooves, forming trans-scale micro/nanostructures. The contact angle decreases with the increase in grid spacing, which may be caused by the increase in the solid–liquid contact area.

**Figure 4:**
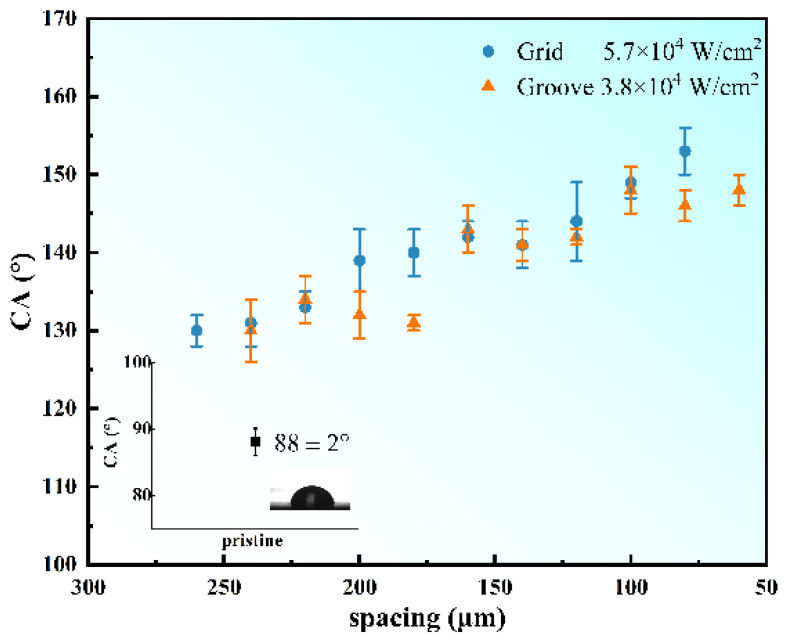
The static contact angles of grid (5.7 × 10^4^ W/cm^2^) and groove (7.6 × 10^4^ W/cm^2^) with spacing from 60 to 260 μm, the inset is the contact angle value and the photograph of pristine CFRP surface.

**Figure 5:**
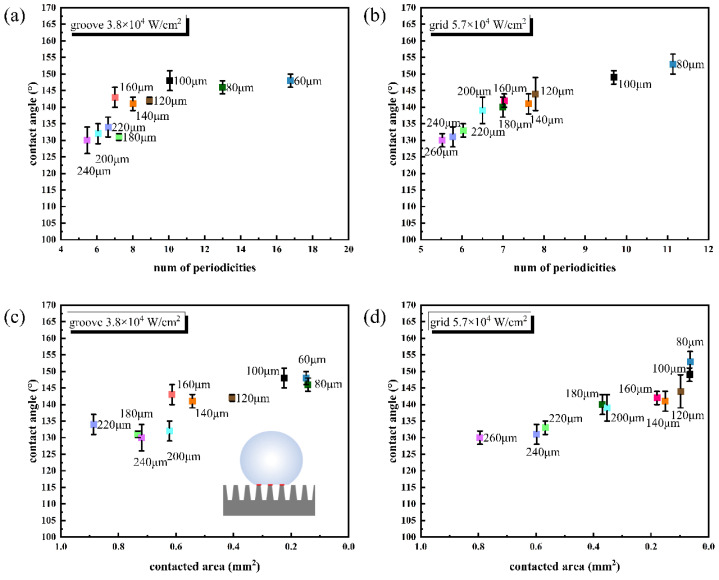
The relationship between number of periodicities and CA on (**a**) groove processed by 3.8 × 10^4^ W/cm^2^, and (**b**) grid processed by 5.7 × 10^4^ W/cm^2^. The relationship of contacted area and CA on (**c**) groove processed by 3.8 × 10^4^ W/cm^2^, and (**d**) grid processed by 5.7 × 10^4^ W/cm^2^.

A larger range (from 60 to 260 μm) of spacing CA figure is shown in Figure 4. The inset of Figure 4 corresponds to the contact angle of the pristine CFRP surface. The contact angle is 88 ± 2°, indicating a typical hydrophilic characteristic. Obviously, there is a steep increase in the contact angle on processed surfaces, indicating a transition from hydrophilic to superhydrophobic. With the decrease in structural spacing, the contact angle increases lineally but with fluctuation. Figure 5a,b shows the relationship between the periodicities number (estimated value) and the CA value of the two patterns. The number of periodicities was expressed as B1/B2, where B1 is the width of the covered area (the area covered by a droplet, not the projected area of a droplet), and B2 is the width of a periodic structure (spacing between the two carved lines). B1 is estimated through the contact angle photograph. In general, with the increase in the number of periodicities, the CA value of the groove structure first increased and then remained stable. Additionally, the CA value of the grid structure increased approximately linearly dependent on the increase in periodicity numbers.

Figure 5c,d shows the relationship between the contacted area (estimated value) and the CA value of the two patterns. Considering that the droplet did not enter the bottom of patterns, but only contacted the convex part of the microstructure (the schematic diagram of the contact area is shown in the inset of Figure 5c), the contacted area was defined as the product of the covered area and the percentage (*f*) of the convex part to a unit area, which is estimated from SEM figure. From Figure 5c,d, though there are inevitable estimation errors, it can be seen that the CA value increases with the decrease in the contact area, reflecting that the CA is strongly dependent on the micro-nano surface structure.

However, as the spacing decreased to 50 μm with other preparation conditions unchanged, the micro convex was damaged, and the surface turned hydrophilic again. Figure S1 showed the optical micrographs and contact angle test pictures of the processed surface with 50 μm spacing. This phenomenon also confirmed that the micro-nano structure, rather than the change of surface chemical states, is the key element to achieve the hydrophilic to hydrophobic transition in CFRP. Considering the CA value of the sample with spacing greater than 200 μm is generally less than 140°, the optimal spacing range was set as 60–180 μm.

Then, a more detailed optimization of process parameters was performed. The laser power density is set from 1.9 × 10^4^ W/cm^2^ to 7.6 × 10^4^ W/cm^2^ with other parameters unchanged (repetition rate = 200 kHz, scanning speed = 100 mm/s). The pattern of groove space from 60 μm to 160 μm and grids space with 80 × 80 μm^2^ to 180 × 180 μm^2^ were written on the CFRP with different laser energy densities.

**Figure 6:**
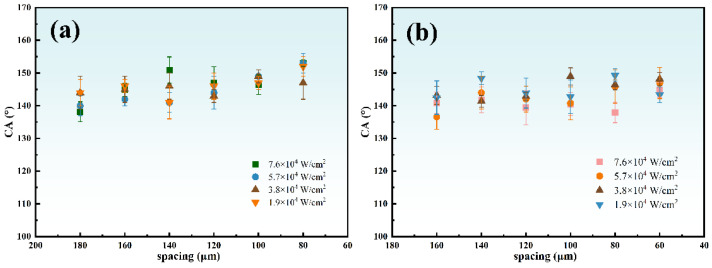
Static contact angles on different surfaces (**a**) groove structure (**b**) grid structure.

Figure 6 shows the static contact angles on the surface of two structures at room temperature. About three samples per parameter were tested and each sample was tested at least five times. The champion value of groove structure is 149 ± 3° (100 μm), and that of the grid structure is 153 ± 3° (80 μm). Therefore, the optimal processing parameters are repetition rate = 200 kHz, scanning speed = 100 mm/s, laser power density = 5.7 × 10^4^ W/cm^2^, and the optimal processing pattern is 80 × 80 μm^2^ spacing grids.

**Figure 7:**
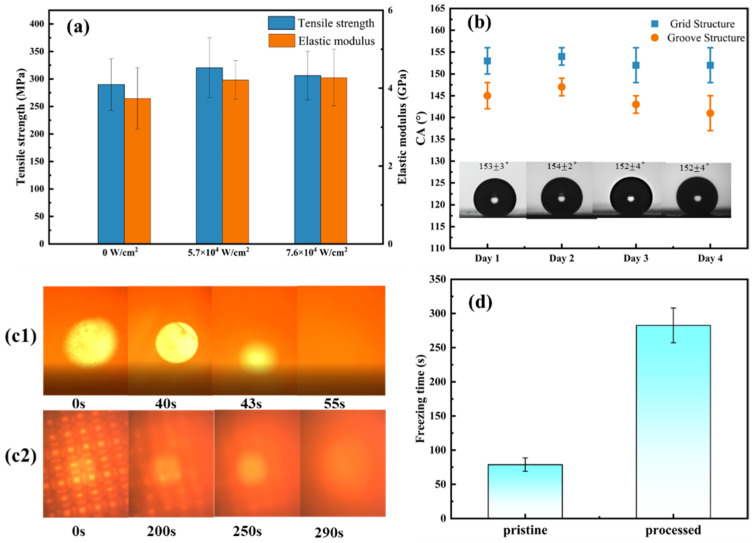
(**a**) Mechanical properties comparison among pristine and 80 μm grid processed by 5.7 × 10^4^ W/cm^2^ and 7.6 × 10^4^ W/cm^2^ laser power density; (**b**) surface contact angle test over 4 days on 80 μm grid and groove patterns; (**c**) CCD camera records of the freezing process of the pristine (**c1**) and 80 μm grid sample processed by 5.7 × 10^4^ W/cm^2^ (**c2**) and (**d**) the corresponding freezing time comparison between pristine and processed at the temperature of −10 °C.

Before further testing, we compared the mechanical properties of the samples before and after processing. Generally speaking, a laser manufacturing process may produce defects on the surface of materials, which may cause a decrease in strength. Only when the mechanical properties are not greatly weakened, the superhydrophobic properties are of practical significance. Then, tensile strength and elastic modulus comparison of pristine and the 80 × 80 μm^2^ spacing grid pattern samples processed by the optimal laser power density of 5.7 × 10^4^ W/cm^2^ and even higher of 7.6 × 10^4^ W/cm^2^ are shown in Figure 7a. The original stress–strain curve is shown in Figure S2. Unexpectedly, the processed samples showed higher tensile strength and modulus than the unprocessed samples. For the unprocessed samples, the tensile strength and elastic modulus are 289.8 ± 27 MPa and 3.7 ± 0.8 GPa, respectively. For the processed samples written by 5.7 × 10^4^ W/cm^2^ laser power density, the values increase to 320.5 ± 54 MPa and 4.2 ± 0.5 GPa. As the laser power density increases, the tensile strength slightly decreases to 306.3 ± 44 MPa, and the elastic modulus is essentially unchanged (4.3 ± 0.7 GPa).

Then, the further surface state stability test and freezing time test were performed. Figure 7b shows the water contact angle changes over 4 days (new droplets were dripped every single test). The blue squares and orange dots present the 80 μm grid and 80 μm groove samples, respectively. The contact angle was measured eight times a day and the average values with the error bars are shown in Figure 7b. The inset in Figure 7b is the corresponding contact angle images of 80 μm grid samples. It can be seen that the contact angle remains stable for 4 days. Additionally, the 80 μm grid patterns were used in subsequent freezing time testing.

The freezing time test was taken on a cooling platform at −10 °C and 28% humidity, and the freezing process where solids come in contact with liquids was recorded by a CCD camera. The CCD camera graphs of the freezing process on the pristine (c1) and 80 μm grid sample processed by 5.7 × 10^4^ W/cm^2^ (c2) are shown in Figure 7c. The moment when the droplet contacts the surfaces were recorded as the beginning and the moment when the spot disappears due to internal scattering of ice is defined as the moment when icing is complete. We attempted to set the camera focal plane at the solid–liquid interface. However, it was hard to set the focal plane accurately. As a result, when the droplet began to freeze, an ice film would first form on the outer layer, which acted like a lens and made the second image (40 s) in Figure 7c1 appear clearer. In Figure 7c2, the processed pattern could be clearly observed through the transparent droplet. However, the picture was blurred and finally faded out because of the internal scattering of ice freezing. From the CCD graphs, the freezing time of a droplet (4 μL) on the processed surface with 80 μm space grids extended from 55 s (on a pristine sample) to 290 s. The statistical data of the freezing time was recorded and are shown in Figure 7d. The average freezing time of pristine is 78 ± 10 s, that of processed surface is 283 ± 25 s, which is four times that of the untreated surface, suggesting a broad prospect in anti-icing and deicing applications.

In order to discuss the relationship of microstructure, contact angle, and freezing time, a detailed theoretical analysis was performed.

**Figure 8:**
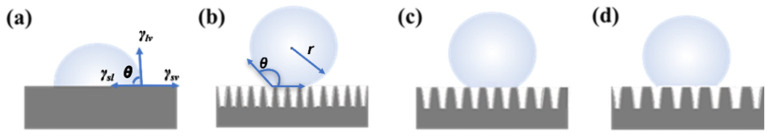
Schematic of a droplet on different surfaces (**a**) pristine, (**b**) fine grids, (**c**) middle grids, and (**d**) large grids.

The wettability model of the raw surface (Figure 8a) can be described by the classical Young’s equation [23]:

(1) $$\cos\theta^{Y}=(\gamma_{sv}-\gamma_{sl})/\gamma_{lv}$$

where $\gamma_{sv}$, $\gamma_{sl}$, $\gamma_{lv}$ represent the surface tension of solid–vapor, solid–liquid and liquid–vapor. $\theta^{Y}$ is the intrinsic contact angle and here is referred to as the contact angle of the untreated CFRP.

Figure 8b is the schematic diagram of droplet morphology on the processed and modified surface. Considering that the surface modification with PFDTS reduces surface energy and droplet adhesion, combined with the actual superhydrophobic characteristic (the contact angle is 153 ± 3°), Cassie state transition was believed to occur [24]. In the Cassie state, an air film exists between the droplet and the sample, which reduces the solid–liquid contact area. In fact, we have observed the suspected air film in the contact angle image shown in Figure S3.

The freezing behavior on the different surfaces can be explained by the freezing nucleation mechanism thermodynamically and dynamically. In the thermodynamic theory, the nucleation and crystallization process of crystals nucleus attached to the surface is a process of liquid–solid phase transformation. According to the heterogeneous nucleation theory, the critical nucleation energy ($\Delta G_{K}$) can be expressed as [25,26]:

(2) $$\Delta G_{K}=\frac{\left(2+\cos\theta \right){\left(1-\cos\theta \right)}^{2}}{4}\left(\frac{4\pi r^{3}\Delta g}{3}+4\pi r^{2}\gamma_{sl}\right)$$

where *r* is the equilibrium radius of the water droplet, and Δg is the difference of the free energy per unit volume between the thermodynamic phase that nucleation is occurring in and the phase that is nucleating. For the given droplet, we suppose that the *r* does not change and the critical nucleation energy $\Delta G_{K}$ is related to two factors, the droplets (Δg) and the surfaces (θ, $\gamma_{sl}$). The greater the nucleation energy, the higher the energy barrier to overcome for the crystal nuclei to form and harder to freeze.

When the droplets are dropped on the CFRP surface, which is put on the cooling platform, direct heat transfer is the main approach for the droplets to overcome the nucleation barrier [27]. Then, the heat transfer rate *ϕ* by Fourier’s law can be written as:

(3) $$\phi =\frac{dQ_{c}}{dt}=-\alpha A\left(T_{2}-T_{1}\right)$$

(4) $$A=f_{s}\pi r^{2}\sin^{2}\theta$$

where *α* is the thermal conductivity coefficient of solid–liquid interface, *Q_C_* is the heat caused by thermal conduction, $f_{s}$ represent the ratio of the convex area to the area covered by the bottom of the droplets, *A* is the actual contact area between the droplet and the material surface (referring to the convex part), *T*_1_ is the original temperature of the droplet, and *T*_2_ is the temperature of the sample surface, which is the temperature of the cooling platform. Equation (3) can be expressed as:

(5) $$\phi =-\alpha f_{s}\pi r^{2}\sin^{2}\theta \left(T_{2}-T_{1}\right)$$

Obviously, the heat transfer rate ϕ is positively related to $f_{s}$ and $\sin^{2}\theta$. When θ > 90°, the larger the θ, the slower the ϕ is. The smaller the $f_{s}$, the slower the ϕ is.

Then, the freezing time *t* can be defined as the time between when the droplet just touches the sample and just overcomes the nucleation barrier. The equation of heat transfer rate *ϕ,* freezing time *t* and critical nucleation energy $\Delta G_{K}$ is as follow.

(6) $$\phi t=\Delta G_{K}$$

(7) $$t=\frac{r\Delta g+3\gamma_{sl}}{-3\alpha \left(T_{2}-T_{1}\right)}\cdot \frac{1}{f_{s}}\cdot \frac{2-\cos\theta -\cos^{2}\theta }{1+\cos\theta }$$

From Equation (7), freezing time *t* is determined by the droplet (r, Δg), the features of solid–liquid interface (surface tension $\gamma_{sl}$ and the thermal conductivity coefficient α), temperature deference ($T_{2}-T_{1}$), surface structure ($f_{s}$) and contact angle (θ). In this work, the experiments were controlled with the droplets, environment, and surface chemical process unchanged, the freezing time *t* was inversely proportional to $f_{s}$, and increased with the increasing contact angle (θ>90°).

Therefore, the surface microstructure can delay icing, and a large number of solid–gas interfaces in the microstructure can reduce the difficulty of deicing. For the sample in Figure 7c2, we adjusted the CCD focal plane to the inside of the droplet and observed the changes in the bubbles inside the droplet during the freezing process. Photos of bubbles were taken and shown in Figure S4, which provided evidence for the increase of the gas–liquid interface in the processed sample.

## 4. Conclusions

In this paper, multi-scale micro-nano structures were processed on aircraft skin materials CFRP for superhydrophobic and anti-icing applications. Grooves and grids with different spacing were carved on CFRP by femtosecond laser processing. The water contact angle increased from 88 ± 2° (pristine) to 149 ± 3° (3.8 × 10^4^ W/cm^2^, groove spacing 100 μm) and 153 ± 3° (5.7 × 10^4^ W/cm^2^, grid spacing 80 μm). The further anti-icing test indicated that the nanostructure of the surface exhibited obvious anti-icing features. The freezing time of a droplet on the pristine surface was 78 ± 10 s, then increased to 283 ± 25 s on the processed surface with 80 × 80 μm^2^ grid spacing. Furthermore, the processed surface achieves superhydrophobic property without deterioration of mechanical properties. This work provides great significance for aircraft active anti-icing technology.

## Supplementary Materials

The following supporting information can be downloaded at: https://www.mdpi.com/article/10.3390/nano12162782/s1, Figure S1. Damaged structure (point array with 50 μm spacing) and its static contact angle. Figure S2. stress-strain curves of (a) the pristine, 80 μm grid processed by (b) 5.7×104 W/cm^2^ and (c) 7.6×104 W/cm^2^. Figure S3. The suspected air film in the contact angle image. Figure S4. CCD camera records of the freezing process (from 258 s to 308 s) of the 80 μm grid sample processed by 5.7×104 W/cm^2^. These pictures record the changes inside the droplet. There are some bubbles observed, which slow down the freezing process.
