# Supplementary material for: Passive Deicing CFRP Surfaces Enabled by Super-Hydrophobic Multi-Scale Micro-Nano Structures Fabricated via Femtosecond Laser Direct Writing
Source: Nanomaterials (Basel). 2022 Aug 13;12(16):2782. doi: 10.3390/nano12162782 (PMC9412491; doi:10.3390/nano12162782)
Supplement: Supplementary file 1 [file nanomaterials-12-02782-s001.zip › nanomaterials-1818318-supplementary.pdf]

Supplementary Material for

Passive Deicing CFRP Surfaces Enabled by Super-Hydrophobic Multi-Scale Micro-Nano Structures Fabricated via Femtosecond Laser Direct Writing

Zihan Zhang [1,†], Jiakang Zhou [1,†], Yuqi Ren [1], Weihan Li [1], Sheng Li [1], Nianyao Chai [1,2], Zhongle Zeng [1], Xiangyu Chen [1], Yunfan Yue [1], Ling Zhou [3,*], Yi-Bing Cheng [1,2], Shuxin Li [1,2] and Xuewen Wang [1,2,*]

1 State Key Laboratory of Advanced Technology for Materials Synthesis and Processing, International School of Materials Science and Engineering, Wuhan University of Technology, Wuhan 430070, China

2 Foshan Xianhu Laboratory of the Advanced Energy Science and Technology Guangdong Laboratory, Foshan 528216, China

3 Hubei Key Laboratory of Theory and Application of Advanced Materials Mechanics, School of Science, Wuhan University of Technology, Wuhan 430070, China

* Correspondence: zling1980@whut.edu.cn (L.Z.); xwwang@whut.edu.cn (X.W.)

† These authors contributed equally to this work.

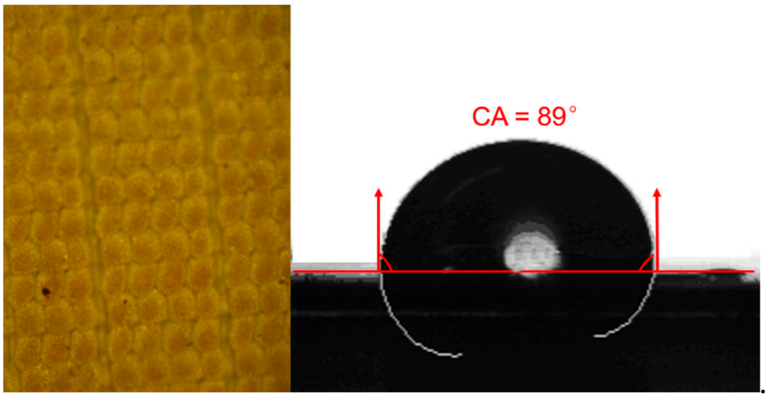

Figure S1. Damaged structure (point array with 50 μm spacing) and its static contact angle.

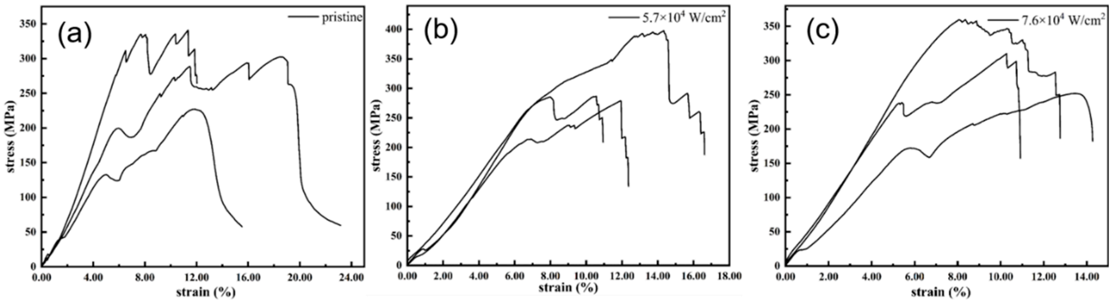

Figure S2. stress-strain curves of (a) the pristine, 80 μm grid processed by (b) 5.7×10^4 W/cm² and (c) 7.6×10^4 W/cm².

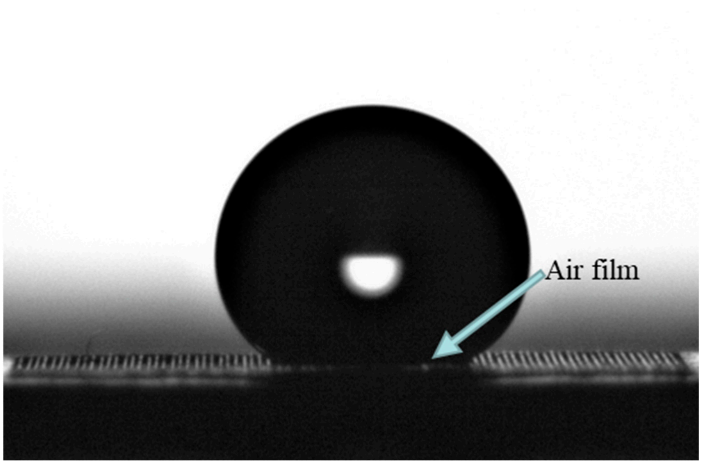

Figure S3. The suspected air film in the contact angle image.

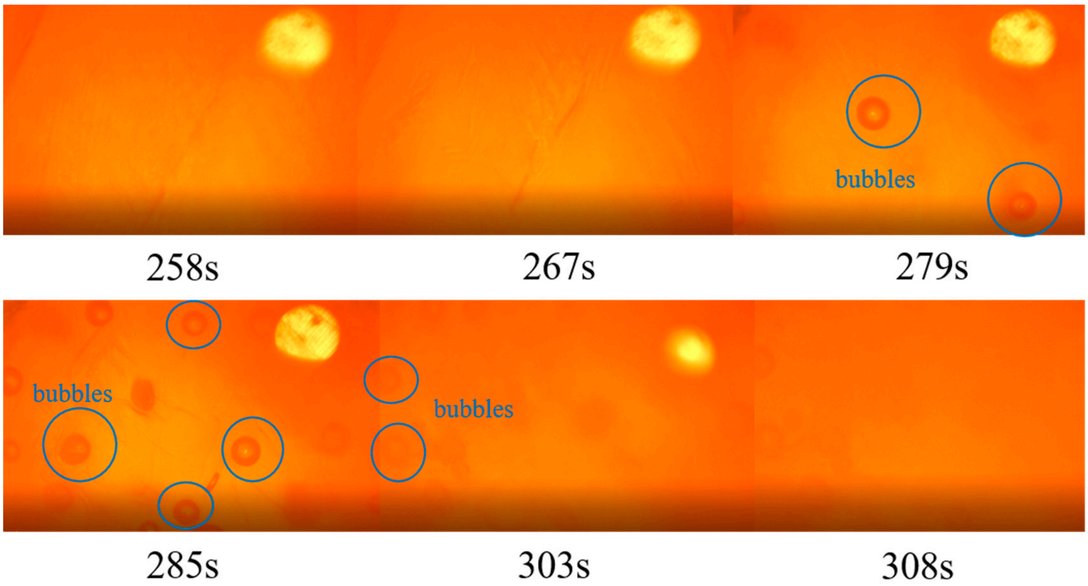

Figure S4. CCD camera records of the freezing process (from 258s to 308s) of the 80 μm grid sample processed by 5.7×10^4 W/cm^2. These pictures record the changes inside the droplet. There are some bubbles observed, which slow down the freezing process.
